# Households willingness to join and pay for community-based health insurance: implications for designing community-based health insurance based on economic Status in Ethiopia

**DOI:** 10.1371/journal.pone.0320218

**Published:** 2025-03-25

**Authors:** Zewdie Birhanu, Morankar Sudhakar, Mohammed Jemal, Desta Hiko, Shabu Abdulbari, Bikiltu Abdisa, Badassa Wolteji Chala, Getnet Mitike, Tigist Astale, Nimona Berhanu

**Affiliations:** 1 Department of Health Behavior, and Society, Faculty of Public Health, Jimma University, Jimma, Ethiopia; 2 Faculty of Public Health, School of Epidemiology and Biostatics, Jimma University, Jimma, Ethiopia; 3 Departmetn of Economics, College of Business and Economics, Jimma University, Jimma, Ethiopia; 4 Departemtn of Statistics, College of Natural Sciences, Jimma University, Jimma, Ethiopia; 5 Department of Economics, Ambo University, Ambo, Ethiopia; 6 International Institute for Primary Health Care, Addis Ababa, Ethiopia; 7 School of Pharmacy, Jimma University, Jimma, Ethiopia; University of Bologna, ITALY

## Abstract

**Background:**

Despite the encouraging results achieved by community-based health insurance in Ethiopia, the program faces significant challenges. Among these challenges is the current practice where premium contributions to Community Based Health Insurance are either a flat rate or based solely on family size, rather than considering households’ socio-economic status The overall aim of this study was to assess households’ willingness to join and pay for Community Based Health Insurance in reference to socio-economic status to design sliding scale-based Community Based Health Insurance contributions in Ethiopia.

**Methods:**

A community based cross-sectional study was conducted in districts from two different contexts: urban areas and agrarian areas in two major regions in Ethiopia, namely Oromia, and Amhara. A double-bounded dichotomous contingent valuation method was used to determine households’ willingness to pay. Descriptive statistics were used to summarize the data. A chi-square test was used to assess background factors associated with willingness to join and pay for Community Based Health Insurance, and tobit regression analyses were conducted to identify factors that determine the amount of willingness to pay for Community Based Health Insurance. The statistical significance of all results was interpreted using an adjusted two-sided Type I error rate of 0.05.

**Result:**

A total of 786 households participated in this study. Overall, 532 (67.7%) study households have ever participated in the Community Based Health Insurance scheme. The reason for never participating was unaffordability of payment (30.3%), and they stated that the service was unsatisfactory (21.7%). Generally, 647 (82.3%) of the households were willing to join Community Based Health Insurance or renew their scheme membership in the future, with higher willingness among rural and urban residents and households with food insecurity (p < 0.05. The average amount households were willing to pay was 538.2 Ethiopian Birr with mode (570.0 Ethiopian Birr). In contrast with the existing premium contribution policy, the vast majority of households preferred premium contributions that considered households’ economic status (81.2%). Increased household size, better household food security, and being rural residents, increased satisfaction with the scheme; and rural households’ economic status significantly predicted the value of money households are willing to contribute to Community Based Health Insurance (p < 0.05).

**Conclusion:**

This study revealed a strong willingness among community members to participate in or renew their membership in the Community Based Health Insurance scheme, with a clear preference for a socio-economic-based sliding scale approach over current flat rate or family size-dependent premium systems. This preference highlights the potential for transforming towards more equitable citizen contributions. Policymakers should therefore consider household economic status, alongside factors like household food security and family size, in determining Community Based Health Insurance membership fees. Furthermore, enhancing the quality of healthcare services is essential to boosting Community Based Health Insurance member satisfaction and ensuring the program’s long-term sustainability. This comprehensive approach not only improves health outcomes but also strengthens community trust and support for the Community Based Health Insurance initiative.

## Introduction

The 2030 Sustainable Development Goals (SDG) emphasizes the importance of primary healthcare in ensuring that all people have access to high-quality healthcare without financial barriers [1]. Sustainable health financing is needed to achieve universal health coverage (UHC), which is a complex process that depends on the availability of resources and the fair distribution of those resources [2, 3]. The interrelated factors that limit UHC are the inadequacy of health resources, overdependence on direct payments by patients, and imbalanced resource utilization [4, 5]. In 2015, 926.6 million people around the world had catastrophic health expenditure (CHE) with a 10% threshold of total consumption, with 109.8 million in Africa [5]. In Ethiopia, a representative survey (2015–2016), indicated that roughly 2.1% of households were affected by CHE, and the largest numbers of affected households were from the Oromia, Amhara, and Southern Nations Nationalities and Peoples (SNNP) regions [6]. In fact, Ethiopia reduced out-of-pocket spending on health from 53% (1995/96) to 31% (2016/17) although this is still significantly higher than the recommended range (15–22%) to safeguard households against CHE costs [7].

The emergence of health insurance is widely acknowledged as one of the most effective means to mobilize local resources for strengthening the financial security of healthcare spending [8, 9]. In line with this, the Ethiopian government commenced CBHI in 2011 intending to maximize domestic resources mobilized for the healthcare industry, boosting health services, and safeguarding society from CHE [10]. In this scheme, enrolment in the program is at the household level, irrespective of family health risks and the socio-economic status of the households [10]. Furthermore, indigent members (those with incomes below the national poverty line) account for approximately 10% of total CBHI membership, and their contributions are directly covered by the government, with the federal government covering 70% of the contribution and the local government covering the remainder [11, 12]. As of 2020, the CBHI program was operating in 80% of the districts in the country, reaching out to all regions and city administrations the level of implementation is quite heterogeneous, ranging from 27% (Gambella) to 76% (in Addis Ababa) [13]. The program has made a significant contribution to improving population health outcomes and mobilizing health resources [14]. It contributed to improving healthcare utilization and reducing socioeconomic disparities in health service utilization between the poorer and wealthier households. Furthermore, CBHI helped improve women’s empowerment and reduce financial barriers to healthcare access [15].

Despite these positive outcomes, the CBHI program has encountered numerous gaps and challenges. In the current scheme, every household contributes an equal amount of premium (flat rate contribution) regardless of their economic status and willingness to pay, which negatively affects CBHI’s financial risk distributional effect (cross-subsidization effects among members); and leads to inequitable citizen contributions to the program. As a result, households with low income may choose to delay or avoid joining the scheme, and households with high wealth status may also be less interested in joining the scheme [16, 17]. Because the better off are paying far less than what they could afford to pay, the CBHI’s limited ability to raise money hinders the program’s sustainability and, ultimately, the effort to establish a fair and equitable health insurance system in Ethiopia. In connection with this, the scheme did not increase the availability of essential inputs to enhance the provision of healthcare services, nor did it increase client satisfaction [15].

Many low-income and middle-income countries (such as Rwanda, Kenya, and Nigeria) that implemented the CBHI program gradually shifted the scheme from the flat rate (fixed premium contribution) to the sliding scheme (premium contribution based on households’ economic capacity) to strengthen their national CBHI programs [8,18,19]. For example, Rwanda’s CBHI scheme strategized the slide rate in such a way that the poor contributed United States Dollar (USD) 3.00 per person, the middle households contributed USD 4.50 per person, and the richer households contributed USD 10.50 per person [20]. The sliding scale contribution scheme helped Rwanda overcome the challenges it faced under the CBHI flat-rate system [20]. People in the sliding payment scheme are more willing to join and pay for health insurance than those in the fixed payment scheme, indicating that socioeconomic payment is an important factor in determining the community’s willingness to pay for the CBHI scheme [21].

In Ethiopia, households’ inability to pay is the most common reason for people dropping out of the scheme, along with other factors such as a lack of medicines at government-run pharmacies and other services [22–25]. To strengthen the national CBHI, the government of Ethiopia has a plan to initiate a new CBHI contribution scheme (shifting from a flat-rate premium payment system to a sliding-scale contribution system) according to the socio-economic status of households. However, the initiative needs to be supported by research evidence to inform the formulation of an acceptable and affordable socio-economic based contribution scale. Yet, local evidence is generally lacking regarding the community’s view on how the sliding scale should be determined and what demographic factors should be considered as additional input to assist the policymakers in determining the amount of premium contribution. Therefore, this study was intended to analyze the community’s experience with the CBHI, its views, and suggestions regarding the basis for premium contributions and its willingness to pay for the CBHI in relation to a sliding scale scheme-based approach.

## Methods and materials

### Study settings

The study was conducted between June 2022 and September 2022 in four rural agrarian districts selected from two major regions of Ethiopia (Oromia and Amhara) and two urban settings, namely Bahir Dar city and Jimma town. The rural districts were Mana and Kersa (from Jimma Zone of Oromia region) and North Mecha and Yilmana Densa (from Amhara region). The population of each district was quite similar in context and livelihood patterns (mainly practicing agricultural activities as a major means of livelihood in rural areas, except for the communities of Mana and Kersa, as they are partly dependent on coffee production and predominantly Muslim in religion.

### Study design and population

The study was a community-based cross-sectional design using a quantitative household survey. The study population was heads of households (spouses in the absence of heads). Households were included in the study regardless of their history of participation in CBHI.

### Sample size and sampling

Sample size was calculated using a single population proportion formula using Epi Info version 7 after considering the following population parameters: the expected proportion of households who were willing to pay (WTP) for the CBHI scheme (p = 89.9%) [23], 95% CI (Zα/2 = 1.96), 3% margin of error, and design effect of two. After considering 5% for non-response, the final sample size was found to be 812 households. The selection of the study communities and households was conducted as follows: First, in each study district/city, two villages (referred to as Kebele in Amhara and Ganda in Oromia) were selected randomly, making a total of 8 rural villages and a total of 4 urban villages. Then, the sample size was distributed to each study community with 63% allocated to rural and the remaining to be sampled from the urban villages. Finally, approximately an equal number of households were sampled from each village through a simple random method using a sampling frame available at the Kebele level. Then, the data collectors approached the selected households for interview and participation in the study.

## Study variables and measurement

### Households’ willingness to join and pay for CBHI

**Fig 1:**
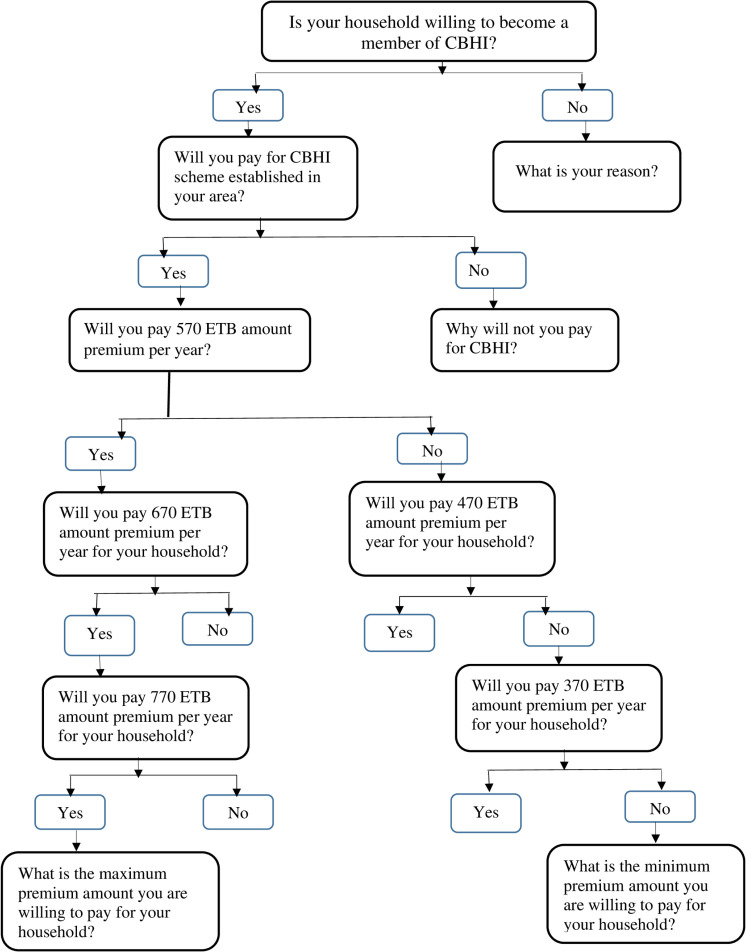
Contingent valuation technique with adjustment of double-bounded dichotomous choice (DC) elicitation.

The main outcome variable, addressed by this study was households’ willingness to join and willingness to pay (WTP) for CBHI scheme. Households’ willingness to join (renewal of membership for those who were active members) CBHI scheme was assessed by asking respondents a key question, “Would your household be willing to become a member of CBHI for the upcoming enrolment?” And the response was recorded as “1” (Yes) and “0” (No). This question was asked all study participants regardless of their membership status at the time of study. On the other hand, WTP is conceptualized as the maximum price a household was willing to pay in exchange for health services, measured using a contingent valuation technique with adjustment of double-bounded dichotomous choice (DC) elicitation method where the first and the second questions were followed by the third question specifying a lower amount, if the answer to the second question were negative, and higher otherwise (Fig 1). The initial Bid for this study was set at 570 ETB, taking into account the premium amounts in the two rural households and towns at the time of the study. At the time of this study, in the Oromia region, the initial premium was 410 ETB for rural households and 500 ETB for taxpayers, whereas in the Amhara region, the initial premium was 350 ETB for rural households and 450 ETB for taxpayers.In addition to considering the existing initial premium amounts, consultations were held with Ethiopian Health Insurance Service (EHIS) stakeholders to determine the initial Bid for this study. The extension of DC elimination to include follow-up question was preferred to give more opportunity for the households, for its incentive compatibility, and more appropriate and practical with population in which literacy level is low [26,27].

### Household wealth index

The wealth index was constructed using households’ ownership of selected assets ranging from television, radio, bicycle, motorbike, phone, refrigerator, car, and possession of a house, livestock animals, farmland, and facilities such as type of floor, toilets, and electricity. Principal component analysis (PCA) was conducted to create a wealth index using the household’s assets, with separate indices for urban and rural areas and categorizations of households were made into three different wealth quintiles of five equal proportions.

### Household food insecurity measures

The Household Food Insecurity Access Prevalence (HFIAP) scale was used, which consists of eight occurrence questions (yes/no) and eight questions about frequency of occurrence over the previous four weeks. The frequency of occurrence of the event was rated as ‘rarely’ (1), ‘sometimes’ (2), and ‘often’ (3). The overall HFIAS score was calculated for each household by summing up responses to each frequency-of-occurrence question; the range of possible scores was 0–24. The higher the score indicates, the more food insecurity the household experiences, and vice versa. Based on the score, categories of food insecurity were defined as food secure (1), mildly food insecure (2), moderately food insecure (3), and severely food insecure (4), as per the standard computation method [28].

### Data collection tools and methods

Semi-structured questionnaire was adapted from literature including the Ethiopian Demographic and Health Survey (EDHS) [29] translated into two local languages (Afan Oromo and Amharic), pre-tested in similar settings, and used for data collection. The questionnaire consisted of different parts including background characteristics of the respondents and households, perceptions, experiences, and satisfaction with CBHI services, food security scale, households’ wealth possession, and willingness to join and pay (S1 File). The questionnaire was administered to household heads by experienced and trained enumerators with a minimum of Level–IV qualification. Adequate training was given to the data collectors, and the research team closely supervised the data collection process.

## Data management and analysis

We used Epi-data version 3.1 for data entry and SPSS version 22.0 for data analysis (S2 Data). Descriptive statistics were computed to summarize the findings. Principal Component Analysis was conducted to compute the wealth index and create a wealth quintile. The chi-squire test was used to assess the association of basic socio-economic factors with a willingness to join and pay for CBHI. The statistical significance of all results was interpreted using an adjusted two-sided Type I error rate of 0.05. Logistic regression and linear regression models were estimated to identify risk factors for the amount of willingness committed to pay for CBHI respectively. Specifically, the logistic regression model estimated is specified as follows:


$$log\frac{P_{i}}{1-P_{i}}=\beta_{0}+\overset{m}{\underset{i}{\sum }}\beta_{m}x_{m}+\varepsilon_{i}$$ (1)

Where the left-hand side variable measures the probability that a household i joins CBHI scheme; β's are the parameters of the model and x's are independent variables which include household level socioeconomic characteristics, regional level, and town/ or village level characteristics, and $\varepsilon_{i}$ is an error term of the model. Likewise, the linear model specifically tobit model was estimated to identify factors associated with the exact amount households are willing to pay. Among candidate linear models, we relied on tobit estimator because the dependent variable, i.e., the exact amount households are willing to pay is censored. This variable is censored with the minimum amount that households are eager to pay 100 ETB (the lower bound) and the maximum possible amount that households are ready to pay 1000 ETB (the upperound). Since some households are not willing to pay at all, we alternatively considered a lower bound of zero.

The tobit model (with lower and upper bound) estimated is specified as follows:


$$Y_{i}^{*}=X_{i}^{'}\beta +\varepsilon_{i}$$

$$\varepsilon_{i}N\left(0,\sigma^{2}\right)$$

$$Y_{i}= \{\begin{array}{c} Y_{i}^{*}ifY_{L}<Y_{i}^{*}<Y_{U}\\ Y_{L}ifY_{i}^{*}<Y_{L}\\ Y_{U}ifY_{i}^{*}\geq Y_{U} \end{array}$$ (2)

Where $Y_{L}$ and $Y_{U}$ are the lower and upper bounds respectively and $Y_{i}^{*}$ is a latent variable that cannot be always observed and Xi's are observable independent variables which include household level socioeconomic characteristics, regional and area specific characteristics among others. For robust model building, we computed six models. As can be seen from Table 8, in Models 1, 3, and 5 the lower bound is 0 while in Models 2, 4, and 6 the lower bound is 100 ETB. In all Models, the upper bound is the same, that is, 1000 ETB.

**Table 1:** Descriptive statistics of Socio-demographic characteristics of the respondent, Sep. 2022.

Variables	Category	Frequency	Percentage
Region	Oromia	438	55.7
	Amhara	348	44.3
Residence/setting	Rural	499	63.5
	Urban	287	36.5
District/City	Kersa	138	17.6
	Manna	129	16.4
	Jimma Town	171	21.8
	Bahir Dar City	116	14.8
	North Mecha	116	14.8
	Yilmana Densa	116	14.8
Sex of respondent	Female	309	39.3
	Male	477	60.7
Age of the respondent	18 – 35	249	31.6
	36 – 53	383	48.6
	>54	154	19.5
Educational status of respondents	No formal education	336	42.7
	Primary (1–6)	163	20.7
	Junior secondary (7–8)	98	12.5
	High school (9–12)	97	12.3
	Diploma	52	6.6
	Degree and above	40	5.1
Marital status of respondent	Married	638	81.2
	Windowed	72	9.2
	Divorced	41	5.2
	Single	35	4.5
Religion of the respondent	Orthodox	384	48.9
	Muslim	385	49.0
	Others^*^	17	2.1
Total family size	1–5	464	59.0
	6–7	213	27.1
	8 and more	109	13.9

* catholic, protestant.

Source: Own compilation (2023)

**Table 2:** Food insecurity among the study households, Sep. 2022.

During the last 4 weeks, how often …	Not at all	Rarely	Sometimes	Often
you worry that your household would not have enough food?	618 (78.7%)	93 (11.8%)	42 (5.3%)	33 (4.2%)
you or any HH member not able to eat the kinds of foods you/they preferred because of a lack of resources?	534(67.9%)	111(14.1%)	87 (11.1%)	54 (6.9%)
you or any household member obligated to eat a limited variety of foods due to a lack of resources?	540 (68.7%)	108(13.7%)	77 (9.8%)	61 (7.8%)
you or any household member have to eat some foods that you really did not want to eat?	677 (86.1%)	45 (5.7%)	37 (4.7%)	27 (3.4%)
any household member has to eat a smaller meal than you felt you needed?	681 (86.6%)	52 (6.6%)	32 (4.1%)	21 (2.7%)
there was ever no food to eat of any kind in your household because of lack of resources to get food?	684 (87.0%)	77 (9.8%)	17 (2.2%)	8 (1.0%)
any HH member go to sleep at night hungry because there was not enough food?	744 (94.7%)	21 (2.7%)	15 (1.9%)	6 (0.8%)
you or any household member go a whole day and night without eating anything?	768 (97.7%)	4 (0.5%)	5 (0.6%)	9 (1.2%)

**Table 3:** Households enrolment status by some independent variables, Sep. 2022.

Variable	Category	Ever enrolled into CBHI, n (%)	Current enrolment status, n (%)
Region	Amhara (n = 348)	225 (64.7%)	211 (60.6%)
	Oromia (n = 438)	307 (70.1%)	295 (67.4%)
Place of residence	Urban (n = 287)	134 (46.7%)	125 (43.6%)
	Rural (n = 499)	398 (79.8%)	381 (76.4%)
Family size	1 – 5 (n = 464)	286 (61.6%)	267 (57.5%)
	6 – 7 (n = 213)	153 (71.8%)	150 (70.4%)
	8 and more (n = 109)	93 (85.3%)	89 (81.7%)
Overall	n = 786	532 (67.7%)	506 (64.4%)

**Table 4:** Participants response regarding adequacy and affordability of CBHI payment, Sep. 2022.

Variables in Category	Response	Do you think the amount you paid is enough to cover your family’s health need?	Do you think the amount you paid is affordable?	Do you think you and your household benefited from CBHI scheme?
Amhara (n = 225)	Yes	149 (66.2%)	105 (46.7%)	174 (77.3%)
	No	52 (23.1%)	100 (44.4%)	51 (22.7%)
	I don’t know	24 (10.7%)	20 (8.9%)	0
Oromia (n = 307)	Yes	114 (37.1%)	148 (48.2%)	240 (78.2%)
	No	155 (50.5%)	130 (42.3%)	67 (21.8)
	I don’t know	38 (12.4%)	29 (9.5%)	0
Urban (n = 134)	Yes	43 (32.1%)	64 (47.8%)	91 (67.9%)
	No	58 (43.3%)	43 (32.1%)	43 (32.1%)
	I don’t know	33 (24.6%)	27 (20.1%)	0
Rural (n = 398)	Yes	220 (55.3%)	189 (47.5%)	323 (81.2%)
	No	149 (37.4%)	187(47%)	75 (18.8)
	I don’t know	129 (32.4%)	22 (5.5%)	0
Overall (n = 532)	Yes	263 (49.4%)	253 (47.6%)	414 (77.8%)
	No	207 (38.9%)	230 (43.2%)	118 (22.2%)
	I don’t know	62 (11.7%)	49 (9.2%)	0

**Table 5:** Satisfaction level of the participant households with CBHI program, Sep. 2022.

Satisfactions with…	Satisfaction level n (%), n = 532				
Very dissatisfied	Dissatisfied	Neutral	Satisfied	Very satisfied
enrollment process	20 (3.8%)	53 (10%)	25 (4.7%)	266 (50.0%)	168 (31.6%)
amount of premium contributed	21 (3.9%)	78 (14.7%)	40 (7.5%)	253 (47.6)	140 (26.3%)
process of premium collection	112 (21.1%)	58 (10.9%)	47 (8.8%)	212 (39.8%)	103 (19.4%)
process of membership card collection	52 (9.8%)	91 (17.1%)	44 (8.3%)	226 (42.3%)	119 (22.4%)
timely access to healthcare	90 (16.9%)	170 (32%)	33 (6.2%)	188 (35.3%)	51 (9.6%)
healthcare package allowed in CBHI	57 (10.7%)	172 (32.3%)	40 (7.5%)	211 (39.7%)	52 (9.8%)
health service provision	94 (17.7%)	185 (34.8%)	36 (6.8%)	180 (33.8%)	37 (7%)
availability of drugs	129 (24.2%)	219 (41.2%)	36 (6.8%)	121 (22.7%)	27 (5.1%)
access to diagnosis service	90 (16.9%)	192 (36.1%)	41 (7.7%)	178 (33.5%)	31 (5.8%)
health staff welcoming and treatment	67 (12.6)	141 (26.5%)	67(12.6%)	215 (40.4%)	42 (7.9%)
quality of service	67 (12.6%)	188 (35.3%)	42 (7.9%)	205 (38.5%)	30 (5.6%)
cost of medical services	105 (19.7%)	149 (28%)	59(11.1%)	187 (35.2%)	32 (6%)

**Table 6:** Households’ willingness to join CBHI scheme in the future, Sep. 2022.

Variable	Category	Proportions willingness to a be member of CBHI/willing	Proportions (among willing to join) willing to pay for CBHI
Yes (n = 786)	Yes (n = 647)
Region^*^	Amhara	266 (76.4%)	244 (91.7%)
	Oromia	381 (87%)	335 (87.9%)
Residence^*^	Urban	212 (73.9%)	172 (81.1%)
	Rural	435 (87.2%)	407 (93.6%)
Total family size	1 – 5	375 (80.8%)	325(86.7%)
	6 – 7	176 (82.6%)	164 (93.2%)
	8 and above	96 (88.1%)	90 (93.75%)
Overall health status perception	Good	617 (82.8%)	554 (89.8%)
	Bad	30 (73.2%)	25 (83.3%)
Wealth quintiles (3 levels)^*^	1^st^ quintile	214 (81.7%)	174 (81.3%)
	2^nd^ quintile	199 (76%)	176 (88.4%)
	3^rd^ quintile	234 (89.3%)	229 (75.3%)
Food insecurity status^*^	Food secure	404 (80.8%)	388 (96.1%)
	Mildly food insecure	82 (90.1%)	72 (87.8%)
	Moderately food insecure	65 (73%)	58 (89.2%)
	Severely food insecure	96 (90.6%)	61 (63.5%)

* significant *at* p < 0.05.

**Table 7:** Study participants’ opinion regarding criteria for determining the amount of CBHI payment, 2022.

How should CBHI payment scale be determined? (n = 786)	Response N (%)	Amhara N (%)	Oromia N (%)	Urban N (%)	Rural N (%)	Overall N (%)
Based on households’ economic status	Yes	280 (80.5)	358 (81.7)	234 (81.5)	404 (82.0)	638 (81.2)
	No	68 (19.5)	80 (18.3)	53 (18.5)	95 (18)	148 (18.8)
Based on households’ family size	Yes	140 (40.2)	188 (42.9)	63 (22)	265 (53.1)	328 (41.7)
	No	208 (59.8)	250 (57.1)	224 (78)	234 (46.9)	458 (58.3)
Based on health status of family members	Yes	14 (4)	54 (12.3)	8 (2.8)	60 (11)	68 (8.7)
	No	334 (96)	384 (87.7)	279 (97.2)	439 (88)	718 (91.3)
should not be determined by any factor Flat scale	Yes	23 (6.6)	12 (2.7)	23 (8)	12 (2.4)	35 (4.5)
	No	325 (93.4)	426 (97.3)	264 (92)	487 (97.6)	751 (95.5)

**Table 8:** Tobit Regression results on predictors of the amount households willing to contribute, Sep.2022.

Variables	Model 1	Model 2	Model 3	Model 4	Model 5	Model 6
Region (Reference = Amhara)						
Oromia	‒221.4^***^	‒196.4^***^	‒513.0^***^	‒470.9^***^	25.9	24.2
	(52.4)	(49.3)	(78.5)	(76.8)	(91.1)	(83.2)
Religion (Ref = Orthodox)						
Muslim	103.4^**^	83.7^*^	253.9^***^	224.7^***^	56.3	37.6
	(50.8)	(47.8)	(73.8)	(72.2)	(78.9)	(71.5)
Protestant	30.4	30.7	276.0	238.3	‒78.5	‒64.1
	(102.3)	(96.2)	(216.4)	(210.5)	(134.0)	(123.0)
Residence (Ref = Urban)						
Rural	140.0^***^	127.6^***^				
	(29.4)	(27.7)				
Age of the Household Head	1.7^*^	1.7^*^	1.5	1.6^*^	2.0	2.0
	(1.0)	(0.9)	(0.942)	(0.9)	(2.6)	(2.4)
Total Family Size	23.8^***^	21.3^***^	5.6	5.7	73.0^***^	64.4^***^
	(5.7)	(5.4)	(5.5)	(5.3)	(14.4)	(13.1)
CBHI Membership in Years	‒6.5	‒5.4	3.5	3.7	‒41.7^**^	‒33.0^**^
	(5.9)	(5.6)	(5.7)	(5.6)	(16.9)	(15.5)
Current Membership (Ref = Yes)						
No	‒1.9	‒15.2	81.1	77.7	‒107.6	‒170.9
	(61.4)	(58.1)	(61.3)	(59.7)	(129.2)	(126.3)
Sick Household Member (Ref = Yes)						
No	7.5	3.5	‒19.8	‒20.6	36.8	34.6
	(25.0)	(23.4)	(23.3)	(22.7)	(63.6)	(57.7)
HFIAS Score	‒29.0^***^	‒26.3^***^	‒17.0^***^	‒16.2^***^	‒18.2^***^	‒17.8^***^
	(2.9)	(2.8)	(3.5)	(3.3)	(6.5)	(6.0)
A Household with chronic illness (Ref=Yes)						
No	31.0	27.7	17.4	17.7	‒23.3	‒34.1
	(23.6)	(22.2)	(23.1)	(22.5)	(58.6)	(53.4)
Satisfaction Score	3.0^**^	3.2^***^	5.4^***^	5.5^***^	8.4^***^	7.4^**^
	(1.2)	(1.2)	(1.3)	(1.2)	(3.2)	(2.9)
Level of Education (Ref = Grade 1‒6)						
Junior/High School-7-12	114.1^***^	108.7^***^	51.1	52.9	208.9^***^	190.2^***^
	(33.4)	(31.3)	(33.2)	(32.3)	(70.3)	(63.7)
Diploma and Above	208.0^***^	200.4^***^	162.7^**^	164.0^**^	314.0^***^	285.7^***^
	(51.9)	(48.6)	(71.1)	(69.0)	(84.6)	(76.5)
No formal education	‒28.7	‒30.4	‒5.4	‒4.6	‒31.9	‒72.0
	(29.4)	(27.5)	(26.6)	(25.9)	(87.3)	(79.7)
Marital Status (Ref = Married)						
Others	‒68.6^**^	‒68.5^**^	‒77.9^**^	‒77.5^***^	‒93.5	‒72.2
	(31.9)	(29.9)	(30.5)	(29.7)	(71.4)	(65.6)
Health Status (Ref = Good)						
Bad	8.2	9.8	22.3	18.8	‒67.1	‒34.5
	(50.090)	(47.0)	(46.0)	(44.7)	(145.7)	(133.3)
Rural Wealth Quintiles (Ref = Quintile 1)			.	.		
Rural Wealth Quintile 2			47.3	46.5		
			(33.1)	(32.2)		
Rural Wealth Quintile 3			122.3^***^	119.9^***^		
			(39.9)	(38.7)		
Rural Wealth Quintile 4			192.4^***^	178.6^***^		
			(48.3)	(47.1)		
Rural Wealth Quintile 5			207.5^***^	194.9^***^		
			(49.2)	(48.0)		
Urban Wealth Quintile (Ref = Quintile 1)						
Urban Wealth Quintiles 2					‒57.6	‒67.9
					(86.9)	(79.7)
Urban Wealth Quintile 3					‒51.6	‒53.5
					(93.3)	(84.9)
Urban Wealth Quintile 4					‒79.8	‒74.9
					(88.9)	(81.4)
Urban Wealth Quintile 5					‒123.8	‒139.9^*^
					(86.3)	(80.8)
Constant	197.2^**^	209.8^***^	358.6^***^	272.0^***^	‒364.8^*^	‒235.4
	(79.1)	(74.2)	(81.4)	(70.3)	(214.5)	(195.1)
N	473	473	346	346	118	118
Censored Observations (Left, Right)	(27,49)	(27,54)	(13,24)	(12,24)	(3,30)	(3,33)

Note: 1:

* ,

** and

*** denote statistical significance at 10%, 5% and 1% respectively 2: Standard errors in parentheses. 3: In Models 1, 3 & 5 lower and upper bounds are 0 and 1000 while 100 and 1000 in other Models

### Ethical considerations

The study was approved by Jimma University institutional review board (IRB) (Ref. No: IHRPGD/418/22) and permission to undertake the study was obtained at each administrative level. Informed verbal consent was obtained from each study participant after explaining the purpose of the study using an information sheet.

## Results

### Background characteristics of the study participants

Seven hundred eighty-six (786) households participated in this study, making a response rate of 96.8%. Accordingly, 438 (55.7%) were sampled from Oromia region and the remaining were from Amhara region. In terms of residence, 348 (63.5%) were recruited from rural areas. Regarding the sex and age of the respondents, 477 (60.7%) of them were males, with the majority in the age range of 36–53 years. Concerning religion, 385(49%) were Muslim and 384(48.9%) were Orthodox Christian (**Table 1**).

### Households’ food insecurity experience and prevalence

Regarding household food insecurity, 252 (32%) of the participants reported that their family members were unable to eat the kind of food they preferred because of a lack of resources, whereas 246 (31.2%) of participants stated that their family members were obligated to eat only limited kinds of food. One hundred sixty-eight (21.3%) of study participants responded that they had a concern that their family members would not have enough food (Table 2). Overall, the household food security score indicated that the majority (63.6%) of households were food secure whereas 11.6%, 11.3%, and 13.5% of the households were mildly, moderately and severely food insecure, respectively.

### Households’ participation in CBHI scheme

Overall, 532 (64.7%) study households were ever participated in CBHI scheme with 225 (64.7%) in Amhara and 307 (70.1%) in Oromia. Rural residents had more participation history than urban residents (79.8% vs 46.7% respectively). With regard to current CBHI membership status, 506 (64.7%) of them were active members of the CBHI at the time of the study (Table 3).

### Reasons for not participating in CBHI scheme

**Fig 2:**
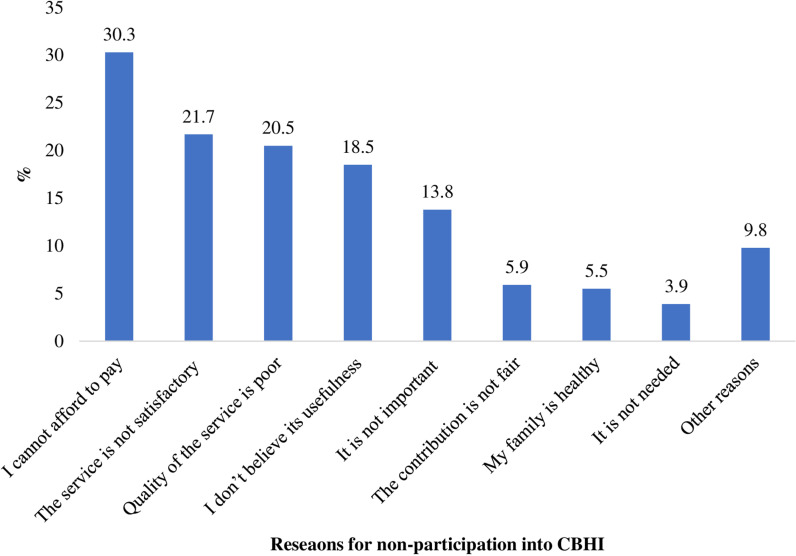
Reasons for not joining CBHI in the past time, Sep. 2022.

Among the participants who never participated in CBHI scheme, a considerable proportion (30.3%) mentioned unaffordability of payment as a reason for not participating, and 21.7% of them stated that services were not satisfactory. Not trusting its usefulness, its importance, and poor service quality were also raised as reasons for not taking part in the CBHI scheme. Other reasons such as being a government employee, not being around during recruitment, and Non Governmental Organization (NGO) employees were also cited as reasons for non-enrolment (Fig 2).

### Adequacy and affordability of the amount of CBHI payment (for households whoever enrolled)

 In households sampled from Amhara region, less than half (105, 46.7%) responded that the premium amount they were contributing for CBHI payment was affordable whereas 100 (44.4%) responded that it was not affordable to them. Similarly, in Oromia region, 148 (48.2%) of the households responded that the amount was affordable to them. A similar proportion of urban residents (47.8%) and rural residents (47.5%) responded that the payment was affordable to them. Regarding the adequacy of the premium contribution to cover their healthcare expenditure, 263 (49.4%) of respondents believed that the amount was sufficient to cover their healthcare costs. Region wise, 66.2% (Amhara) and 37.1% (Oromia) of the households believed that the amount they were contributing was enough to cover their families’ health needs. Regarding the benefits of being CBHI member, more than three-quarters of participants from both regions responded that they benefited from CBHI program (Table 4).

### Satisfaction level with CBHI scheme

Around half of the study participants (50.0%) who ever participated in the CBHI scheme responded that they were satisfied with the enrolment process, whereas 41.2% (219) responded that they were dissatisfied with the unavailability of prescribed medicines in health facilities. The amount of premium contributed, the method of collecting premium, and the membership card collection process are the items with a high level of satisfaction among the satisfaction questions raised by respondents (Table 5). The mean satisfaction of participants with CBHI program was 37.13 ±  10/30, with quite similar levels of mean satisfaction scores in both regions (36.8/60 in Amhara region and 37.4/60 in Oromia region). Similarly, there was no much difference in the level of satisfaction among rural and urban residents (37.6/60 and 36/30 respectively) (Table 5). The overall mean satisfaction was 37.1 ± 10, with no significant variations by region (36.8 ±  10 in Amhara, 37.4 ±  10 in Oromia) and by residence as well (36 ±  11.8 in Urban and 37.6 ±  9.3 in rural).

### Willingness to join or renew membership for CBHI scheme

Overall, 647 (82.3%) of the households were willing to join or renew membership of CBHI scheme in the future. Participants from Oromia region showed a higher willingness to join CBHI than the participants from Amhara region, 381 (87%), 266 (76.4%) respectively. Rural residents showed more willingness to join CBHI program than urban residents (87.2% and 73.9% respectively). These differences were statistically significant (p < 0.05). Higher family size was also associated with increased willingness to join/renew their membership (Table 6).

### Households’ willingness to pay for CBHI scheme

 Of those who were willing to join/renew membership of the CBHI scheme (n = 647), 579 (89.4%) were willing to pay for the premium, and the rest responded that they would not pay for the program. Rural residents were slightly more willing to pay for the program (81.1% and 93.6% respectively). Food-secure households were more willing to pay than severely food-insecure households (96.1% and 63.5% respectively) (Table 6).

### Reasons for declining to join CBHI membership

**Fig 3:**
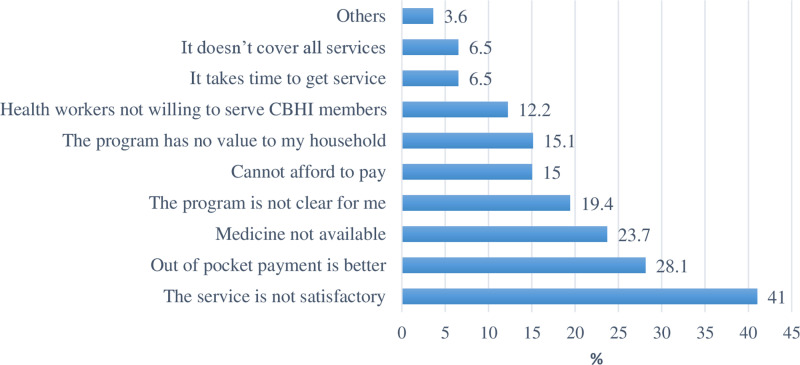
Reasons for not joining CBHI program in the future, Sep. 2022.

Among the study participants who responded they would not participate in CBHI in the future, 57 (41%) of them admitted they had no plans to join the CBHI because of the inadequate care they received from medical facilities, while 39 (28.1%) of them admitted that out of pocket payment is better (Fig 3).

### Premium amount households willing to pay for CBHI

**Fig 4:**
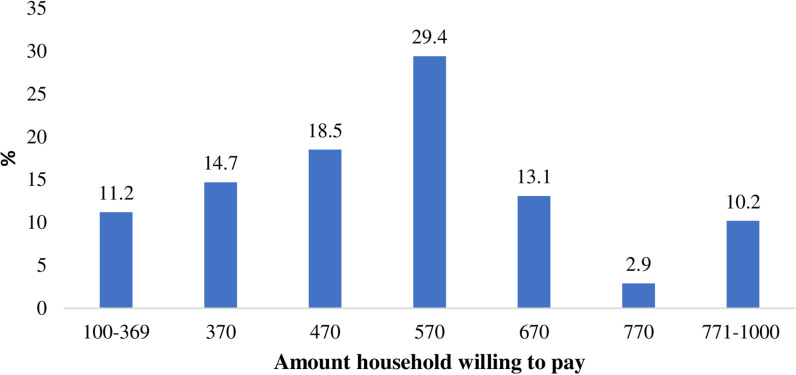
Willingness to pay for CBHI program (after overlapping Bid response corrected), Sep.2022.

Of those who were willing to join the scheme, 322 (41.0%) were willing to pay the first Bid (570 ETB/year) at the household level. For the upstream follow-up, 152 (19.3%) and 76 (9.7%) agreed that they could pay 670 ETB and 770 ETB, respectively. Of respondents who responded they could pay 770 ETB, 31 (3.9%) of them mentioned 1000 ETB as the maximum amount they could pay, and the remaining were mentioned between 770 ETB and 1000 ETB. On the downstream, among the participants who responded ‘**No**’ to the initial Bid (570 ETB) and 107 (13.6%) 85 (10.8%) of them reported that they could pay 470 ETB and 370 ETB, respectively. After the overlapping Bid response was corrected, the distribution of amounts households willing to pay is shown in Fig 4. Limiting the analysis only to those who were willing to pay and overlapping Bid corrected, the average amount households willing to pay was 538.2 ETB with Standard Deviation (SD) of 190 ETB and mode of 570.0 ETB. Region wise, the average was 575.8 ETB in Amhara, 510.8 ETB in Oromia, 532.8 ETB in Urban areas, and 540.7 ETB in rural areas.

### Participant households’ opinions regarding the amount of additional payment for additional family members

Study participants were asked open-ended questions for their opinion regarding the amount of additional payment for additional family members with ≥ 18 years and additional wifives. Accordingly, 706 (89.6%) responded that only 100 ETB or less should be added for additional family members with ≥ 18 years, and the remaining stated over 100 ETB

### Participant households’ opinions on how premium contribution should be determined

Households were asked regarding what factors should be considered to determine the amount of premium contribution, and 638 (81.2%) responded that the payment should be based on households’ economic level, whereas 328 (41.7%) responded based on family size (Table 7).

### Predictors of households willing to pay the necessary amount for CBHI program

Tobit regression results showed that there was a positive association between household size and the amount households were willing to pay keeping all other predictors constant. This can be seen from Models 1, 2, 5, and 6, where the coefficients are consistently significant (Table 8).

For example, for an increase in family size by one member, there was a 23.75 ETB increase in the predicted value of money a household was willing to contribute keeping all other predictors constant (Model 1). Likewise, when family size increases by one member, the predicted value of money a household was willing to pay increases by 21.33 ETB (Model 2), 72.98 ETB (Model 5), and 64.40 ETB (Model 6) keeping all other factors constant. On the contrary, there was a strong negative association between households’ food insecurity status and their willingness to contribute to CBHI. More specifically, with a unit increase in food insecurity score, the predicted value of money a household was willing to contribute to CBHI would decrease by 28.55 ETB (Model 1), 26.28 ETB (Model 2), 16.99 (Model 3), 16.20 (Model 4), 18.18 (Model 5) and 17.8 2 (Model 6). On the other hand, a rural household was willing to contribute about 140 Birr more to CBHI than an urban household (Model 1) and about 128 ETB more to CBHI than an urban household (Model 2).

In addition, the analysis shows that there is a strong positive association between satisfaction scores and households’ willingness to contribute. Specifically, a unit increase in satisfaction score is associated with an increase in the predicted value of money households are willing to pay by 2.98 ETB (Model 1), 3.19 ETB (Model 2), 5.37 ETB (Model 3), 5.53 ETB (Model 4), 8.42 ETB (Model 5) and 7.43 ETB (Model 6). The results show a strong relationship between households’ satisfaction with the services provided by the scheme and their willingness to pay. Similarly, there is a strong and positive association between rural households’ economic status and the exact amount they willing to pay. This can be seen in models 3 and 4. Households in the 5^th^, 4^th^ and 3^th^ quintiles are willing to contribute more money to CHBI in comparison to households with lower economic status, namely, the 1^st^ quintile. However, there is no statistically significant difference between rural households within the lowest economic category namely, Quintile 1 and Quintile 2 in terms of their contributions to CBHI scheme. Within urban households, there is no association between households’ economic status and the exact amount they are ready to contribute to CBHI.

## Discussion

In this study, we assessed and analyzed households’ willingness to join and pay for the CBHI scheme as well as their experiences and satisfaction with it in selected urban and rural villages in Ethiopia. One of the strong success indicators in the CBHI program is the community’s participation, and participation should be sustainably measured by the renewal rate [30]. This metric is pivotal as the program’s effectiveness hinges on its ability to enroll a broad spectrum of beneficiaries to pool resources effectively and provide financial protection to those in need [31]. In our study, only 67.7% of randomly sampled households had ever participated in the CBHI scheme, with a significant portion showing intermittent or inconsistent participation when examining the frequency of enrollment. Despite the initiation of CBHI in Ethiopia in 2011 [32], the number of households reporting multiple enrollments remains relatively low. In Ethiopia, the CBHI was initiated in 2011 [32], but the number of households reporting a higher number of participation was quite low. Despite the program’s aim to provide financial security and access to essential healthcare for the community, community involvement has fallen short of expectations. This limited participation can be attributed to several factors, including an inequitable contribution system, limited subsidies for the poorest households, and inadequate improvements in healthcare quality. The premiums required by the scheme have also been relatively modest compared to rising healthcare costs, potentially leaving enrolled individuals with inadequate financial protection. Consequently, individuals with fewer health needs and those from more affluent households may lose interest in joining CBHI, adversely affecting the program’s effectiveness for the poorest households. One national study in Ethiopia indicated that, on average, 28% of the CBHI members did not renew their membership [15], and another study reported a discontinuation rate of 17.32% [33], suggesting that inconsistent participation and high membership dropout from the CBHI scheme are persistent challenges. These findings highlight persistent challenges of inconsistent participation and high dropout rates within the CBHI scheme.

Nevertheless, it is important to note that of those who ever enrolled in the scheme, only 64.7% of them are current members of CBHI, implying that 35.3% of those who had a history of participation dropped out from the scheme during the study period. Interestingly, the unaffordability of the premium contribution was the major reason cited for non-participation (never participation) in the program. To realize the existing premium contribution amount, all households in rural Oromia were contributing a flat rate of 410 ETB per household per year, and it didn’t depend on any factor including households’ socio-economic status, whereas in rural Amhara, the contribution was based on family size, with households with a larger family size contributing a higher premium amount per year (350 ETB for families of size 1–5; 400 ETB for families of size 6–7; and 480 ETB for families of size>= 8). Likewise, for urban residents, the contribution was 510 ETB (flat rate) for Jimma town residents (study community) but stratified for the residents in Bahir Dar based on family size (400 ETB ETB (1-5 family size); 450 ETB (6-7 family); and 550 ETB (above 8 families). It is important to note that a flat rate contribution may affect the poor section of the community, as they may not afford to pay, and that dependence on family size for charging a higher premium contribution without considering households’ socio-economic status is also problematic and inequitable, because, large families are more common among the poor community members [29].

Experiences from countries where Community-Based Health Insurance (CBHI) schemes have been implemented, such as Rwanda [20], demonstrate that a flat rate contribution model often results in households with lower incomes choosing to delay or avoid enrollment, thereby creating inequities in financial contributions to the scheme.

In this study, a significant majority of households (81.2%) expressed a preference for CBHI premium contributions to be based on socio-economic status, with nearly half (41.7%) suggesting that family size should also be considered in determining premium amounts. Interestingly, a higher proportion of rural households advocated for family size consideration compared to urban residents (53.1% vs. 22%) for determining premium rates.

Currently, in rural areas, households are required to contribute an additional 20% of the basic premium for each member above the age of 18. The majority (over 80%) indicated willingness to pay between 100–200 ETB per person per year for this additional coverage, which is higher than the current 20% rate.

The suggestion to consider both socio-economic status and family size is logical as it promotes equitable financial contributions to the scheme. This proposal contrasts sharply with current practices, which either employ a flat rate or base premiums solely on family size, and is rejected by less than 5% of the study population. The flat rate contribution scheme could significantly impact both households and the healthcare system. Ignoring disparities in household economic status may disappoint the poorest and middle-income households, reducing participation rates and undermining one of the key goals of CBHI: ensuring equal access to healthcare without financial barriers. Additionally, the flat rate system fails to mobilize sufficient funds because it does not adjust premiums based on household economic status. An equity-based contribution system could increase

Experiences from countries such as Rwanda, Kenya, and Nigeria show that sliding-scale premium contributions based on economic status are successful in strengthening the CBHI system’s financial viability and reinforcing equity in citizens’ financial contributions and protection [18–20]. Rwanda, in particular, changed its flat rate scale in 2011 to a sliding scale based on households’ economic status (creating CBHI broad categories of destitute households, medium households, and upper households). Premium rates were different per household in each category based on their economic status, enabling coverage to increase from less than 7% of the CBHI target population in 2003 to 74% in 2013 [20]. The finding from the current study is in favor of the lessons from countries where the CBHI scheme has been recognized internationally for its success, and Ethiopia has a lot to learn from these experiences. Our evidence informs us that transforming the flat rate scheme to economic status based on the premium (slide scale) with potential consideration of family size and level of household food insecurity status (i.e., inverse relationship with willingness to pay) is a basic formula to determine the CBHI premium to be contributed by households. The negative relationship between household food insecurity and willingness to pay is intuitively appealing as food insecure households are forced to allocate a lion’s share of their budget to food and food-related items and hence they might not have enough money left over to contribute to the CBHI scheme. Thus, there is an urgent need to develop a standardized methodology for premium contributions across cities and regions in Ethiopia to ensure the optimal implementation of the program. This transformation would likely enhance coverage, improve financial protection, promote equity in citizen contributions to the scheme, and ensure the sustainability of the CBHI program.

The extent to which households are willing to pay for the health insurance premium and the amount of the declared price for the exchange of services are important indicators for estimating how much each economic category of the household should contribute voluntarily [21,34]. Designing customized insurance benefit packages is essential to ensure that the costs remain manageable within available resources, thereby minimizing the risk of financial strain [35]. The fundamental feature of the CBHI program is that it targets the community by pooling funds to offset the cost of healthcare and, thus, targets the community’s willingness to participate, which is a critical determinant factor for the continuity of the program. In this study, households’ willingness to join the scheme was quite good (82.4%) with a relatively higher willingness rate in Oromia (76.4% in Amhara vs 87% in Oromia) and rural settings (73.9% in urban vs 81.1% rural). Perhaps, this could be because households in the urban centers might prefer out-of-pocket payment to cover their healthcare expenditure due to better access to and availability of health services. The level of willingness to join demonstrated in the present study is consistent with several earlier local studies that reported willingness to join ranging from 73.6% to 90.9% [36–42], with a few exceptions where two studies reported willingness to join rates of 60.5% [43] and 65% [44]. A systematic review also reported a pooled prevalence of 78% willingness to join, which is close to the findings from the present study. The persistence and consistency of people’s willingness to join the program may reflect growing support and acceptance of the CBHI, which has important implications for the program’s sustainability and ownership if well translated into policy actions.

In the current study, many factors such as place of residence, wealth, and food security status were positively related to increased willingness to join the program. This study also identified similar predictors associated with households’ willingness to contribute to the CBHI scheme. Earlier studies also documented that household income, family size, the household head’s education, household health status, household’s age or being older, membership in any association, a history of illness, perceptions of the quality of health services, trust in the scheme and the health system, and increased awareness about the scheme are factors that are significantly associated with WTP for the scheme) [9,37–39,22,45–47].

In the present study, the average willingness to pay was 538.2 ETB, with relatively higher in the Amhara region (575.8 ETB vs. 510.8 ETB) than in the Oromia region, and more than half (56.4%) were accepted to pay 570 ETB or more, which is a clear indication that there is a mounting interest and willingness from the community to contribute even more premium than they are currently paying (blow 570 ETB). Remarkably, there were significant numbers of households that were declared to pay even higher amounts, such as much as 1000 ETB annually. Earlier local literature reported a wide range of average household willingness to pay for the scheme per year, from 211 ETB to 500 ETB [9,38,39,41,44,46]. The average willingness to pay reported in the present study is relatively higher compared to previously reported average amounts of willingness to pay, which might be related to many factors such as increased peoples’ expectations for a higher premium rate combined with increased inflation in Ethiopia; and might also be linked to people’s realization of the insufficiency of the amount they are currently paying for the scheme.

In the present study, a significant portion of households (38.9%) expressed the belief that their current premium contributions were insufficient to cover their family’s healthcare needs. This perception likely reflects a growing awareness of the financial shortfalls or deficits faced by the healthcare system due to the limited premium amounts being collected. Consequently, there appears to be a developing willingness within the community to contribute more. These findings underscore the need to reasonably increase premium contributions, taking into account factors such as households’ wealth status and family size to ensure adequate per house or per-person per-year contributions.

In several previous studies, wealth status and family size were identified as important determinants for consideration in the determination of premium contribution [22,23,37,38,44,45,46,48]. Literature also documented additional factors that should be considered to enhance people’s willingness to pay, such as increasing people’s awareness, their positive attitude about the scheme [ 22,37,49], perceived access to public health facilities [37, 38], their membership in any associations [23,44,45], their trust in the scheme’s management, and their households’ perceptions of the quality of health service [46].

If this interest is properly translated into policy action, it provides a great opportunity for the CBHI to generate more revenue, thereby contributing to the viability and sustainability of the program. This critical analysis of the current research evidence, supported by benchmarks from other countries, mainly Rwanda, shows that there is a high need for Ethiopia to move away from the flat rate scheme towards a sliding scale based on family size and households’ economic status for stratification of households at least into four categories (indigent, lower, middle, and upper). The indigent will be subsidized by the government (following current policy practices) but with different contributions of price per person per year (PPPY) for the different economic categories. Yet, it is essential to keep compulsory enrolment at the household level (as supported by the recent CBHI policy), not individual membership. This is because, when the contribution is set at an individual level, the household may limit the number of household members enrolled (at the agreed-upon PPPY price PPPY) to reduce the total cost per household for the insurance.

Interestingly, rural residents exhubuted a relatively higher level of willingness to pay than urban residents (540.7 ETB vs 532.8 ETB) indicating a lower acceptance of the scheme in urban areas. In fact, the willingness to join the CBHI scheme was also significantly lower in urban areas. This disparity may stem from various factors, including the recent implementation of the program in urban areas, its limited integration into urban communities, weaker community engagement, and differing perceptions among urban residents (e.g., perceptions that the scheme is unnecessary or that they can access healthcare through other means). Addressing these issues requires a well-designed qualitative exploratory study to explore the barriers and underlying motivations or lack thereof for joining and paying for the CBHI scheme in urban settings in Ethiopia

Furthermore, this study identified several important factors that negatively affect households’ engagement in the CBHI program. These include poor quality and unsatisfactory services, a lack of belief in the importance and usefulness of the program; dissatisfaction with the services rendered by the scheme, and unfair contributions to the scheme. To strengthen and ensure the sustainability of the CBHI, it is crucial and equally important to address critical pulling factors. This is because people’s motivation and willingness to join and contribute to the scheme could be easily reversed if the system and supply side [50] (in terms of improving quality, access, supplies, and availability) could not respond to the growing demand and expectations from beneficiaries. Earlier studies also adequately documented widespread clients’ bad experiences with the scheme, dissatisfactions [51,52] with health services (both in quality and access/availability) [53, 54]; unavailability of drugs and supplies; distrust of health care providers and the scheme [ 53, 55]; poor receptions from health workers and management; and shortage of supplies [56] are critical deterring factors.

### Strengths and limitations of the study

The present study aimed to include residents from various locations across Ethiopia’s two largest regions, ensuring representation from both urban and rural areas. This approach enabled us to capture diverse views, perspectives, and experiences, potentially enhancing the generalizability of our findings beyond the current setting. Additionally, this study represents a pioneering effort in assessing the community’s preferences for structuring CBHI premium contributions based on economic categories and considering family size—a novel approach in the field. However, the present study lacks qualitative insights, prompting future research to explore households’ perspectives on socio-economic-based contributions to the CBHI scheme.

## Conclusions

The present study indicates that the community’s willingness to participate in the CBHI scheme is encouraging; with clear readiness and motivation to make the necessary premium payment for the scheme, and a higher declared willingness to pay compared to previous research findings. This reflects that there is a potential demand, for the community-based health insurance scheme, expressed through a higher premium commitment. The greatest implication of the present finding is that there is a clear indication (as preferred by the majority of the respondents) for the CBHI premium contribution scale to be based on the socio-economic status of the households with consideration of family size for deciding the premium price per household per year. As demonstrated by the present study, households’ wealth quintile along with considerations of important factors, particularly family size, and household food security status, with urban stratification gives a realistic approach to creating a sliding (socio-economic-based categories) scale CBHI scheme for Ethiopia. Hence, to strengthen CBHI’s financial viability, and sustainability and reinforce equity in citizens’ financial contributions, CBHI stakeholders, particularly the Ethiopian health insurance agency and regional health bureaus, are required to move away from the flat or non-standardized premium scale and instead design a simplified sliding premium scale based on the socio-economic status of the households. This effort should be supported by local research evidence in addition to the effective engagement of key stakeholders and the community at all levels in the entire process. Furthermore, it is opportune for the government to initiate pilot testing and scale up the implementation of the socio-economic-based sliding scale CBHI system across Ethiopia.

## Supporting information

S1 File (DOCX).

S2 Data (SAV)

## Abbreviations

CBHI: Community-Based Health Insurance
CHE: Catastrophic Health Expenditure
DC: Dichotomous Choice
EDHS: Ethiopian Demographic and Health Survey
EHIS: Ethiopian Health Insurance Service
HFIAP: Household Food Insecurity Access Prevalence
NGO: Non Governmental Organization
PCA: Principal Component Analysis
PPPY: Price Per Person Per Year
RWF: Rwandan Franc
SD: Standard Deviation
SDG: Sustainable Development Goal
SNNP: Southern Nations, Nationality and people
SPSS: Statistical Package for the Social Sciences
UHC: Universal Health Coverage
USD: United States Dollar
WTJ: Willingness to Join
WTP: Willingness to Pay.
