# Supplementary material for: Households willingness to join and pay for community-based health insurance: implications for designing community-based health insurance based on economic Status in Ethiopia
Source: PLoS One. 2025 Mar 25;20(3):e0320218. doi: 10.1371/journal.pone.0320218 (PMC11936286; doi:10.1371/journal.pone.0320218)
Supplement: S1 File — (DOCX). [file pone.0320218.s001.docx]

Jimma University

**Household Survey Questionnaire**

**Respondent: Head of the household, if the head is unavailable second most responsible person from the family**

**Introduction:** Good morning/afternoon. My name is _____________________. I have come from Jimma University to collect data on the research that is aimed at understanding communities’ perceptions, experiences, willingness, and needs regarding the payment system for health insurance from the community. This research aims at. I am here to request you to participate in this research. What we learn from this study will be used to design a simplified sliding scale Community-Based Health Insurance (CBHI) contribution rate based on households’ economic status in Ethiopia. This will help avoid/reduce some of the community concerns and challenges related to payment for health insurance.

**Procedures/Risks/Compensation:** We do not think there are any risks associated with participating in this study other than the possibility that some topics may make you feel slightly uncomfortable talking about. However, you can refuse to answer certain questions if you are not comfortable, or decline to participate in the interview at all. Your honest answers will help the researchers understand important issues that could benefit communities and the health system in Ethiopia in general. We are going to visit up to 900 households in different districts in three regions of Ethiopia, both in rural and urban areas, including pastoral communities. The interview will take about _____________hours/minutes

**Confidentiality**: All of your responses will be kept confidential and I will not collect any information that could connect your identity to the information you share.

Your thoughts and opinions are valuable, and I would be very thankful to have you participate in this interview.

**Contacts:** If you have any questions or concerns at any time related to this study you may contact:

Dr Zewdie Birhanu, Principal Investigator, Jimma University.

Telephone: +251-917025852

**Do you agree to participate in this study?**

1. Yes……. [continue the interview]
2. No…….. [ End the interview]

Name of interviewer________________Signature________________Date____________

Interview time: |____|____|

Checked by: _________________________________________

Date of interview (dd/mm/yyyy) |____|____**|_2_|_0_| _2_|_2 |**

**Section I: Socio-demographic Characteristics**

| **No.** | **Questions and filters** | **Coding category** |
| --- | --- | --- |
|  | Name of region | ______________[insert] |
|  | Name of City | ______________[Insert/NA] |
|  | District Name | ______________[insert] |
|  | Kebele name | ______________[insert] |
|  | Goti/Gare | ______________[insert] |
|  | Setting | 1. Urban 2. Rural |
|  | Sex of the respondent | 1. Female 2. Male |
|  | Relationship of the respondent to family? | 1. Wife (spouse)  2. Husband  3. Family member |
|  | How old are you? *(in completed years)* | ______________ |
|  | Education status of respondent? | 1. Primary (1-6) 2. Junior secondary (7-8) 3. High school (9-12) 4. College diploma/ TEVET 5. University degree and above 6. No formal education |
|  | What is your current marital status? | 1. Single 2. Married (married living together) 3. Married (living separately) 4. Widowed 5. Divorced |
|  | What is your religion? | 1. Orthodox 2. Islam 3. Protestant 4. Catholic 5. Others *(Specify)___________________________* |
|  | Your household size (usual members) | Male: _____________  Female: ____________  People age >1yrs:___________ |
|  | Do you have another wife (for male respondent) or do your husband) have another wife (for female respondent)? | 🗖 Yes how many___________________  🗖 No |

**Section II. Household livelihood strategy and incomes**

1. We would like to learn about the economic activities of your household is participating in. **What is the main economic activity of your household?**
   1. Livestock production/rearing ……………………..🗖 Yes 🗖 No
   2. Agricultural farming like crop farming……………..🗖 Yes 🗖 No
   3. Small business/private company………………….....🗖 Yes 🗖 No
   4. Shop/market/trader…………………………………..🗖 Yes 🗖 No
   5. Causal work/daily laborer………………….……....🗖 Yes 🗖 No
   6. Government employ …………………………….…..🗖 Yes 🗖 No
   7. NGO employee ……………………………………...🗖 Yes 🗖 No
   8. Others (specify)____
2. **Household non-cash activities**
   1. Does the household have farming land? 🗖 Yes 🗖 No
   2. Does your household own non-farming land? 🗖 Yes 🗖 No
   3. Does your household own irrigation land 🗖 Yes 🗖 No
   4. Does your household own a house? 🗖 Yes 🗖 No
   5. Does your household cultivate crops? 🗖 Yes 🗖 No
   6. If your household cultivates crops, which types of crops does your household often cultivate
      - 1. Maize/corn 🗖 Yes 🗖 No
        2. Beans 🗖 Yes 🗖 No
        3. Sorghum 🗖 Yes 🗖 No
        4. Teff 🗖 Yes 🗖 No
        5. Wheat 🗖 Yes 🗖 No
        6. Barely 🗖 Yes 🗖 No
        7. Coffee 🗖 Yes 🗖 No
        8. Fruit/vegetable 🗖 Yes 🗖 No
        9. Chat 🗖 Yes 🗖 No
        10. Others_________________
3. Does your household live private house or rental? 🗖 own/private 🗖 rental
4. Does this household own any livestock and/or poultry? 1. Yes 0. No
5. If yes to Q18, which of the following animals does this household own?

| - 1. Oxen | 🗖 Yes | 🗖 No |
| --- | --- | --- |
| - 1. Milking cow | 🗖 Yes | 🗖 No |
| - 1. Calf | 🗖 Yes | 🗖 No |
| - 1. Other cattle (bull, heifer, oxen) | 🗖 Yes | 🗖 No |
| - 1. Goat | 🗖 Yes | 🗖 No |
| - 1. Sheep | 🗖 Yes | 🗖 No |
| - 1. Camel | 🗖 Yes | 🗖 No |
| - 1. Mule | 🗖 Yes | 🗖 No |
| - 1. Donkeys | 🗖 Yes | 🗖 No |
| - 1. Horse | 🗖 Yes | 🗖 No |
| - 1. Laying hens | 🗖 Yes | 🗖 No |
| - 1. Non-laying hens | 🗖 Yes | 🗖 No |
| - 1. Chickens | 🗖 Yes | 🗖 No |
| - 1. Pullet | 🗖 Yes | 🗖 No |
| - 1. Beehives | 🗖 Yes | 🗖 No |

**Section III. Perceptions and experiences: related to CBHI**

|  | Have ever heard or know about health insurance services? | 🗖 Yes 🗖 No→Q41 |  |
| --- | --- | --- | --- |
|  | Have you ever enrolled in a health insurance program? | 🗖 Yes 🗖 No→33 |  |
|  | How long since you become a member of the health insurance program? | ____________[insert in years] |  |
|  | How many times did you get enrolled? | ___________[insert number] |  |
|  | Of these enrolments, how many times your enrolment was voluntary? | ___________[insert number] |  |
|  | Currently, is your household a member of health insurance? | 🗖 Yes 🗖 No→Q33 |  |
|  | How many members of the household are enrolled or covered by the insurance currently? | 🗖 enrolled (number):__________  🗖 non-enrolled (number): __________ |  |
|  | Is your current enrolment into the program voluntary? | 🗖 Yes 🗖 No |  |
|  | How much did you pay to be a member of a household in your recent membership? | For registration: _____ETB  For annual contribution: _________ETB  Total _________ETB |  |
|  | Do you think this amount (mention total contribution) of contribution is fair considering your household economic status? | 🗖 Yes 🗖 No 🗖 DK |  |
|  | Do you think this amount is an adequate contribution to cover the health needs of a family? | 🗖 Yes 🗖 No 🗖 DK |  |
|  | In your opinion, do you think the amount you paid to join health insurance is affordable to your household? | 🗖 Yes 🗖 No |  |
|  | If No Q31, how much annual payment do you think is affordable to your household? | _____________[Insert amount] →Q34  ______________DK→Q34 |  |
|  | What was the reasons for not being enrolled into health insurance?  Multiple responses possible | 🗖 Don’t believe it is useful  🗖 Not important  🗖 🗖Not necessary  🗖 My family is healthy  🗖 Can’t afford to pay  🗖 Difficult to get service even when enrolled  🗖 Quality of services is poor  🗖 Contribution is not fair  🗖 Others_______________________________ |  |
|  | Do you know how much the family/ household should contribute to become a member of health insurance? | _____________[Insert amount] |  |
|  | Do you think you/your household benefited from being a member of health insurance? | 🗖 Yes 🗖 No |  |
|  | Do you think the amount should depend on household family size? | 1. 🗖 Yes 🗖 No |  |
|  | Do you think the amount of annual contribution for health insurance should consider the income level of the household? | 🗖 Yes 🗖 No |  |

**Section IV: Satisfaction with CBHI services**

|  | **To what extent are you satisfied with CBHI services…?** | **5** | **4** | **3** | **2** | **1** | **NA** |
| --- | --- | --- | --- | --- | --- | --- | --- |
|  | Enrolment process | 🗖 | 🗖 | 🗖 | 🗖 | 🗖 | 🗖 |
|  | Paying premium/amount of money contributed | 🗖 | 🗖 | 🗖 | 🗖 | 🗖 | 🗖 |
|  | Payment collection methods | 🗖 | 🗖 | 🗖 | 🗖 | 🗖 | 🗖 |
|  | Membership card collection process | 🗖 | 🗖 | 🗖 | 🗖 | 🗖 | 🗖 |
|  | Timely access to healthcare when visit health facilities | 🗖 | 🗖 | 🗖 | 🗖 | 🗖 | 🗖 |
|  | Health service packages allowed for members | 🗖 | 🗖 | 🗖 | 🗖 | 🗖 | 🗖 |
|  | Health services provisions in your area/facilities | 🗖 | 🗖 | 🗖 | 🗖 | 🗖 | 🗖 |
|  | Availability of medicine/drugs at health facilities | 🗖 | 🗖 | 🗖 | 🗖 | 🗖 | 🗖 |
|  | Access to diagnostic facilities/laboratory services | 🗖 | 🗖 | 🗖 | 🗖 | 🗖 | 🗖 |
|  | Health staffs welcoming and treatment | 🗖 | 🗖 | 🗖 | 🗖 | 🗖 | 🗖 |
|  | Quality of health services | 🗖 | 🗖 | 🗖 | 🗖 | 🗖 | 🗖 |
|  | Cost of medical services | 🗖 | 🗖 | 🗖 | 🗖 | 🗖 | 🗖 |

5=strongly satisfied, 4=satisfied, 3=neutral/no idea, 2=less satisfied, 1=strongly dissatisfied

**Section V: Family Health Conditions**

|  | Was there any person who was sick from any illness in your household during the last month? | 1. Yes 2. No |  |
| --- | --- | --- | --- |
|  | From which types or levels of health facility does your family often seek care when a family member gets sick?  Multiple responses possible | 1. Health post  2. Health Centre  3. Hospital  4. Pharmacy  5. Private clinic/hospital  6. Other (specify) |  |
|  | In a typical care-seeking for medical treatment, how much money do you spend at a private clinic or hospital | ___________[amount] |  |
|  | In a typical care-seeking for medical treatment, how much money do you spend at a governed facility? | - - - 1. At Health centre: _______       2. At Hospital: _________ |  |
|  | Is there any member of the household who is sick from any chronic illness (name each disease for the respondent to remind them)?  Hypertension 🗖 Yes 🗖 No  Diabetes 🗖 Yes 🗖 No  Heart diseases 🗖 Yes 🗖 No  Kidney diseases 🗖 Yes 🗖 No  Cancers 🗖 Yes 🗖 No  Chronic gastritis 🗖 Yes 🗖 No  HIV/AIDS 🗖 Yes 🗖 No  Allergy (e.g. Sinusitis) 🗖 Yes 🗖 No  Disability 🗖 Yes 🗖 No  Blindness 🗖 Yes 🗖 No  Back pains 🗖 Yes 🗖 No  mental problems 🗖 Yes 🗖 No |  |  |
|  | Overall, how do you rate the overall health conditions of your family? | 🗖 excellent  🗖very good  🗖good  🗖poor  🗖very poor |  |

**Section VI: Household Assets and Facilities**

| 1. **Does your household have the following items?** | | | |
| --- | --- | --- | --- |
|  | **Household asset** | **Response** | |
|  | Electricity? | 1. Yes | 0. No |
|  | Watch/clock? | 1. Yes | 0. No |
|  | Radio/tape | 1. Yes | 0. No |
|  | Television | 1. Yes | 0. No |
|  | Mobile telephone | 1. Yes | 0. No |
|  | Non-mobile telephone | 1. Yes | 0. No |
|  | Refrigerator | 1. Yes | 0. No |
|  | Table | 1. Yes | 0. No |
|  | Chair | 1. Yes | 0. No |
|  | Bed with cotton/sponge/spring mattress | 1. Yes | 0. No |
|  | Electric mitad | 1. Yes | 0. No |
|  | Kerosene lamp/pressure lamp | 1. Yes | 0. No |
|  | Bicycle | 1. Yes | 0. No |
|  | Motorcycle | 1. Yes | 0. No |
|  | Animal-drawn cart | 1. Yes | 0. No |
|  | Car | 1. Yes | 0. No |
|  | Water supply | 1. Yes | 0. No |
|  | Toilet | 1. Yes | 0. No |
|  | kitchen | 1. Yes | 0. No |
|  | gas/electric stove | 1. Yes | 0. No |
|  | sewing machine | 1. Yes | 0. No |
|  | House in town | 1. Yes | 0. No |
|  | Livestock | 1. Yes | 0. No |
|  | Blanket/Gabi | 1. Yes | 0. No |
|  | Water storage pit | 1. Yes | 0. No |
|  | Jewels (Gold and silver) | 1. Yes | 0. No |
|  | Axe | 1. Yes | 0. No |
|  | Machete (Gejera) | 1. Yes | 0. No |
|  | Pasture land | 1. Yes | 0. No |
|  | Crop field | 1. Yes | 0. No |
|  | Coffee plant | 1. Yes | 0. No |
|  | Beehives | 1. Yes | 0. No |
|  | Chickens | 1. Yes | 0. No |
|  | Sickle (Machid) | 1. Yes | 0. No |
|  | Riffle/Gun | 1. Yes | 0. No |
|  | Shelf | 1. Yes | 0. No |

**Section VII: Household food security**

I am going to ask you a few questions about food in your household. [Note: Rarely= once or twice in the past 4 weeks, Sometimes= 3-10 times in the past 4 weeks, Often= more than 10 times in the past 4 weeks]

| S/N | Question | Response Options |
| --- | --- | --- |
|  | In the past four weeks, did you worry that your household would not have enough food? | Yes, 0. No→Q50 |
|  | How often did this happen? | 1 = Rarely  2 = Sometimes  3 = Often |
|  | In the past four weeks, were you or any household member not able to eat the kinds of foods you preferred because of a lack of resources? | Yes, 0. No→Q52 |
|  | How often did this happen? | 1 = Rarely  2 = Sometimes  3 = Often |
|  | In the past four weeks, did you or any household member have to eat a limited variety of foods due to a lack of resources? | Yes, 0. No→Q54 |
|  | How often did this happen? | 1 = Rarely  2 = Sometimes  3 = Often |
|  | In the past four weeks, did you or any household member have to eat some foods that you did not want to eat because of a lack of resources to obtain other types of food? | Yes, 0. No→Q56 |
|  | How often did this happen? | 1 = Rarely  2 = Sometimes  3 = Often |
|  | In the past four weeks, did you or any household member have to eat a smaller meal than you felt you needed because there was not enough food? | Yes, 0. No→Q58 |
|  | How often did this happen? | 1 = Rarely  2 = Sometimes  3 = Often |
|  | In the past four weeks, was there ever no food to eat of any kind in your household because of a lack of resources to get food? | Yes 0. No→Q60 |
|  | How often did this happen? | 1 = Rarely  2 = Sometimes  3 = Often |
|  | In the past four weeks, did you or any household member go to sleep at night hungry because there was not enough food? | Yes 0. No→Q62 |
|  | How often did this happen? | 1 = Rarely  2 = Sometimes  3 = Often |
|  | In the past four weeks, did you or any household member go a whole day and night without eating anything because there was not enough food? | Yes 0. No→Q64 |
|  | How often did this happen? | 1 = Rarely  2 = Sometimes  3 = Often |

**Section VIII: Willingness to join and pay for CBHI scheme in the future (WTP)**

| Q/N | Question | Response category |
| --- | --- | --- |
|  | Would your household be willing to become a member of CBHI next year? | - 1. Yes→64   2. No →63 |
|  | What is/are your main reason/s? | _________________________ |
|  | Will you pay for health insurance established in your area (regardless of amount)? | 1. Yes 2. No→74 |
|  |  |  |
|  | As a household, will you pay a 770 ETB amount premium to become a health insurance member? | 1. Yes 2. No→75 |
|  | What is your maximum willingness to pay for CBHI? | ____________[insert amount] |
|  | Will you pay 670 ETB? | 1. Yes 2. No |
|  | Will you pay 570 ETB? | 1. Yes 2. No |
|  | Will you pay 470 ETB? | 1. Yes 2. No |
|  | Will you pay 370 ETB? | Yes  No |
|  | What is your minimum willingness to pay for health insurance? | ___________[insert amount] |
|  | According to the government policy, any household member age greater than 18 years should pay an additional contribution to get enrolled in health insurance along with the family member. For this, how much will you pay an additional contribution for each person? | ___________[insert amount] |
|  | According to the government policy, a husband having more than one wife should pay an additional contribution to get enrolled in health insurance along with the family member. For this, how much will be an additional contribution for the second wife? | ___________[insert amount] |
|  | Why will you not pay for health insurance? | 1. The program has no value to my household 2. We cannot afford to pay 3. The government should pay for such a program 4. Other members of the society should pay 5. Out-of-pocket payment is better 6. I am not clear about the proposed program 7. Quality of services is poor 8. Health workers do not accept insurance services 9. Other (specify)___________ |
|  | If there is an option of paying in kind (commodities), will your household contribute? | 1. Yes 2. No |
|  | In your view, how should the contribution to health insurance made?  **Multiple responses possible** | 1. Considering the family size of households 2. Considering the economic/income level of households 3. Considering the health status of households 4. Shouldn’t be dependent on any factor or condition; just equal contribution |
|  | Economically better households should contribute more while poor households contribute less | - - - 1. Yes 2. No |
